# Supplementary material for: Development of chemokine network inhibitors using combinatorial saturation mutagenesis
Source: Commun Biol. 2025 Apr 3;8:549. doi: 10.1038/s42003-025-07778-6 (PMC11969024; doi:10.1038/s42003-025-07778-6)
Supplement: Supplementary file 4 — Supplementary Data 2 [file 42003_2025_7778_MOESM4_ESM.zip › SupplementaryData3/scripts/xattune_ci.tar!/./script/analysis/JK/JK_singlemetaanalysis_plot.pdf]

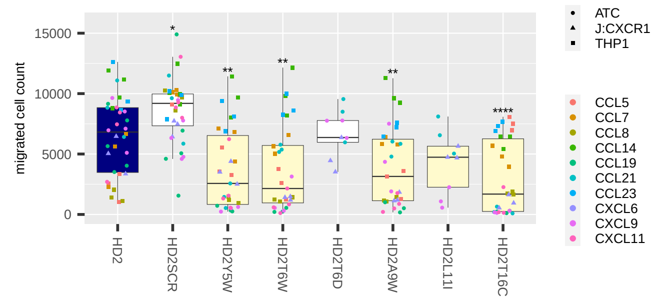

migrated cell count
0
5000
10000
15000
HD2
HD2SCR
HD2Y5W
HD2T6W
HD2T6D
HD2A9W
HD2L11I
HD2T16C
*
**
**
**
****
ATC
J:CXCR1
THP1
CCL5
CCL7
CCL8
CCL14
CCL19
CCL21
CCL23
CXCL6
CXCL9
CXCL11
